# Getting Started in Text Mining: Part Two

**DOI:** 10.1371/journal.pcbi.1000411

**Published:** 2009-07-31

**Authors:** Andrey Rzhetsky, Michael Seringhaus, Mark B. Gerstein

**Affiliations:** 1 University of Chicago, Chicago, Illinois, United States of America; 2 Yale University, New Haven, Connecticut, United States of America; Princeton University, United States of America

We are, in a sense, drowning in information. Today, it is unusual for scientists even to read a journal cover to cover—much less to personally parse all information pertinent to even a narrow research area. Increasingly complex content, large digital supplements, and a staggering volume of publications are now threatening old-fashioned scientific reading with extinction. But by using computers to sift through and scour published articles, the nascent technology of text mining promises to automate the rote information-gathering stage—hopefully leaving to human minds the more challenging (and rewarding) activity of higher thinking.

This article is intended to continue where Cohen and Hunter [1] left off in “Getting Started in Text Mining,” an introduction in the January 2008 issue of *PLoS Computational Biology* which covered the actual mining of text and its digestion into small quanta of computer-manageable information (http://www.ploscompbiol.org/doi/pcbi.0040020). In this overview of the field, we begin by summarizing the major stages of current text-processing pipelines. We now focus on the downstream questions scientists can ask using text-mining and literature-mining engines. At times, we (deliberately) blur the boundary between today's approaches and tomorrow's possibilities.

**Figure 1:**
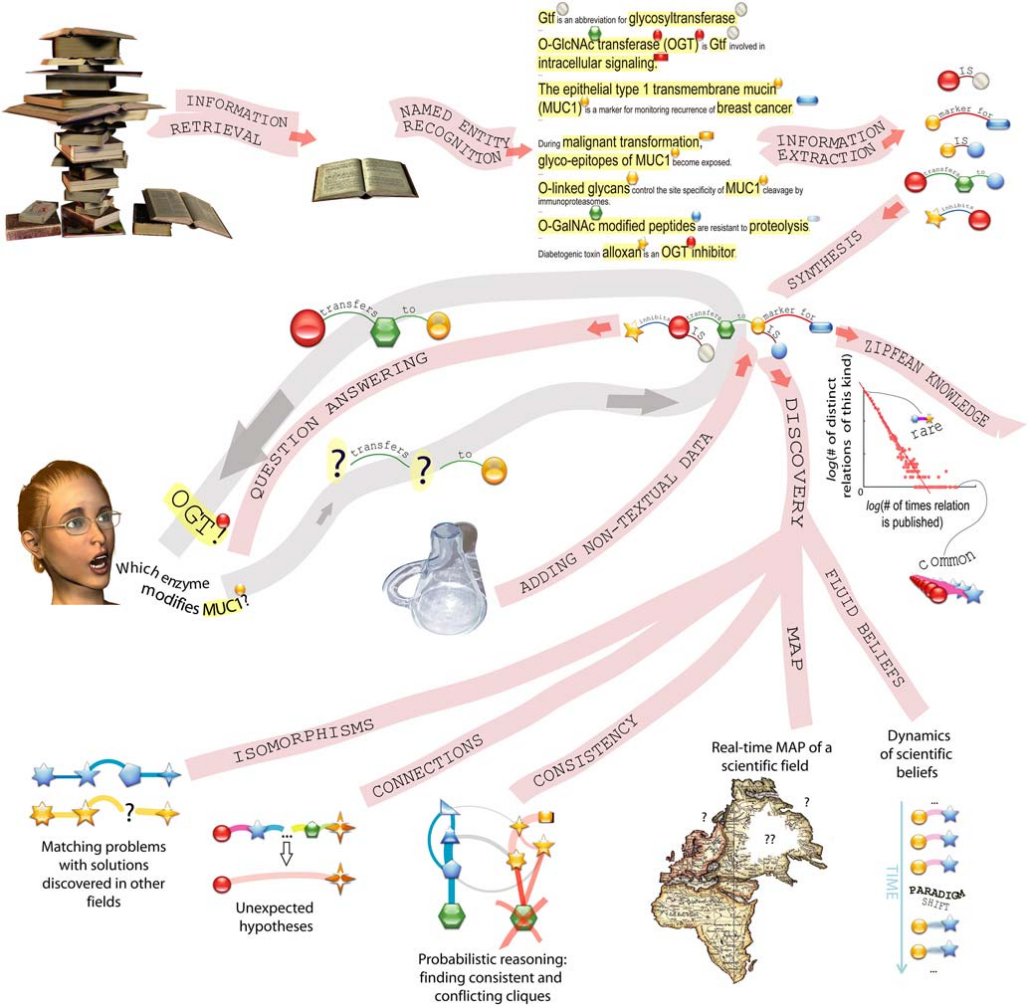
Major techniques and applications of text mining. It is common to divide the task of text mining into information retrieval, named-entity recognition, and information extraction. Extracted information can be further used for building systems for answering questions, fusing experimental data with literature-derived information, implementing computational creativity (discovering esoteric connections between facts, matching solutions in one field with open problems in another one, capturing cliques of internally consistent observations that are inconsistent across cliques), and analysis of large-scale dynamics of scientific fields.

Figure 1 shows a high-level overview of the stages in text mining, with a focus on its applications. We begin at the top left of the figure, which shows the process of information retrieval—how we select relevant documents [2]. Unfortunately, free full-text access remains impossible for a large portion of scientific journals. In some fields, such as chemistry, even article abstracts are inaccessible for a large-scale analysis. The obvious outcome is that articles published in open-access journals have a better chance of being identified as relevant hits than others appearing in traditional “closed-access” journals. Electronic access to text obviously impacts all stages of text mining.

Once the documents have been chosen by an information retrieval engine, a computer scans the text and picks out the various entities (objects, concepts, and symbols) in each sentence. This process, called named-entity recognition [3], draws upon dictionaries of synonyms and homonyms, in addition to machine-learning tools [4], so that an individual entity (say, a protein) is recognized consistently—even though it may be referred to by several different names and acronyms [5]. Named-entity recognition is closely related to the design of controlled terminologies [6] and ontologies for the annotation of texts and experimental data [7]—a process often requiring a monumental community effort [8].

The next step is information extraction (IE) (see pp. 545–559 in [9]). Here, entities are assembled into simple phrases and clauses that capture the meaning of the mined text. To accomplish this, two or more entities are juxtaposed, and meaningful action words—called *predicates*—are chosen to link the entities. For instance, we might say gene X *genetically interacts with* gene Y, or protein A *binds to* protein B. Each completed clause describes a basic relationship between entities. The question then becomes, what can we do with all these simple or complex clauses?

The answer is, quite a lot—which helps explain why text mining is poised to become a powerful central pillar in scientific research and recordkeeping. The lower two-thirds of Figure 1 illustrates how the results of information extraction (IE) can be synthesized and used.

Because IE yields a collection of phrases linking entities through predicates, one of its simplest but valuable uses is to answer simple questions posed to an automated system [10]. In this approach, human questions are digested by a linguistic engine (likely using the same process as employed on original mined text) and mapped to simple phrases. These question phrases are then queried against the database of phrases already stored in the computer, which were generated through the application of IE to analyzed text. (Another mode of question answering, bypassing generation and querying of a database entirely, involves *direct* search and analysis of relevant texts. These texts can be stored at a local computer disk or distributed on numerous computers around the world.) Figure 1 outlines the basic process by which the machine interprets the question, queries its database of stored relationships, and returns an answer.

IE-generated knowledge often tracks closely the needs of experimental biologists. Typical IE systems are developed in direct response to acute practical problems, such as large-scale annotation of regulatory regions in genomes [11], collecting published claims about experimental evidence supporting a collection of assertions [12], and condensing sparse information about phenotypic effects of mutations in proteins [13].

Of course, IE-generated databases can be supplemented with additional data gleaned from experiment, or contributed through other non–text-mining means. A simple user interface could facilitate contributing raw experimental data or other information into the database of relationships expressed as simple phrases—again, entities linked by actions (see, for example, the REFLECT system, http://reflect.ws/). Adding more such data should correspondingly increase the effectiveness of the computer's answers to user questions.

Another major use for the database of IE-generated phrases is to employ the collection itself for the discovery of new information [14],[15]. One approach to this is to seek out “idea isomorphisms”, by which we mean identifying similar types of logical constructs across different contexts. Finding that similar small ideas (or phrases) occur in different fields might allow researchers to bridge different areas of inquiry. Such bridging of fields, in turn, might uncover new connections, thereby suggesting new and unexpected hypotheses that can then be tested experimentally.

The collection of phrases can also be used to vet and prune itself by examining the consistency among many entries. For instance, conflicting or erroneous data can be flagged. By examining each record situated within a large number of records, the preponderance of evidence could assist in identifying and resolving errors. Say, for example, that 20 distinct phrases all indicate that protein A interacts with protein B, and one phrase suggests otherwise; we might probabilistically argue, then, that the lone conflicting statement is false and should be disregarded—unless it is supported some other way.

An additional approach to using these phrases—in a mega-scale fashion—is to construct a “map of science”, a global description of the interrelationships between different fields of inquiry. This is similar conceptually to PubNet [16], which highlights connections between authors. However, the map of science would be generated not through coauthor relationships but through clustering the underlying scientific fact claims themselves, as represented in the IE phrase collection. To do this, researchers would cluster papers according to their IE-derived phrase content; any two papers can be compared in this way to derive a measure of their similarity and overlap in terms of information content. By repeating this process, researchers could create a distance map of all papers in science, and, along the way, of all the factoids that the information content of the papers themselves comprise.

In addition, researchers might track the changing nature of the IE phrases over time to examine the dynamics of scientific belief. This could involve observing as simple phrases themselves change in occurrence or content over time, or we might watch these simple ideas and truth claims crop up in the scientific literature and track their development that way.

Finally, the middle right-hand section of Figure 1 depicts a very simple type of analysis involving the IE-generated simple phrase collection. This approach involves simply looking at the phrases' occurrence in the databases, and recording which statements tend to occur more than others. This type of analysis normally generates a kind of power law–type structure, where it becomes apparent that a few phrases occur many times, but most others only occur a few times.

Text/literature mining is a powerful approach, one we expect to substantially bolster the scientific reporting and discovery process in coming years. Applying the organizational, storage, and pattern-matching capabilities of modern computers to the vast corpus of scientific information contained in the literature (present, past, and future) will not only transform the vast archives of science into rapid-access searchable computerized data, but no doubt also catalyze the discovery of much new knowledge. We hope that this brief “getting started” report highlights some of the major and promising avenues opening as a result of advances in text mining.

*Note to the reader:* The field of text mining is young and growing rapidly, and our own interests and experiences have in large part shaped our perspective on it. We are constrained by length limits here to (reluctantly) omit several topics, such as text mining in conjunction with image analysis, important community text-annotation efforts, and ontology engineering—each important in its own right. Furthermore, every issue touched upon in this essay comes with a rich diversity of views and approaches in the text-mining community. While we cannot possibly do justice to this complexity, the reader should reject the impression that there is but a single correct way to perform text analysis.
